# Aberrant DNA methylation in melanoma: biomarker and therapeutic opportunities

**DOI:** 10.1186/s13148-017-0332-8

**Published:** 2017-04-04

**Authors:** Goran Micevic, Nicholas Theodosakis, Marcus Bosenberg

**Affiliations:** 1 Department of Dermatology, Yale University School of Medicine, New Haven, CT 06520 USA; 2 Department of Pathology, Yale University School of Medicine, New Haven, CT 06520 USA

**Keywords:** Epigenetics, Melanoma, DNA methylation, DNMT, Azacitidine, Biomarker, DNMT1, DNMT3B

## Abstract

Aberrant DNA methylation is an epigenetic hallmark of melanoma, known to play important roles in melanoma formation and progression. Recent advances in genome-wide methylation methods have provided the means to identify differentially methylated genes, methylation signatures, and potential biomarkers. However, despite considerable effort and advances in cataloging methylation changes in melanoma, many questions remain unanswered.

The aim of this review is to summarize recent developments, emerging trends, and important unresolved questions in the field of aberrant DNA methylation in melanoma. In addition to reviewing recent developments, we carefully synthesize the findings in an effort to provide a framework for understanding the current state and direction of the field. To facilitate clarity, we divided the review into DNA methylation changes in melanoma, biomarker opportunities, and therapeutic developments. We hope this review contributes to accelerating the utilization of the diagnostic, prognostic, and therapeutic potential of DNA methylation for the benefit of melanoma patients.

## Background

Aberrant DNA methylation is an epigenetic hallmark of melanoma, and many studies suggest it plays an important in both melanoma formation and progression [1]. Recent advances in genome-wide methylation methods have provided the means to rapidly identify differentially methylated genes, methylation signatures, and potential biomarkers in melanoma. However, despite considerable effort and advances in cataloging methylation changes in melanoma, many questions remain unanswered. Here, we discuss the role of DNA methylation in gene silencing, its potential for biomarker development and as a therapeutic target, including the potential to enhance responses to immune therapies in melanoma.

### Approaches to identifying differentially methylated DNA

New discoveries in melanoma epigenetics have been driven by a continually improving set of tools useful for interrogating the epigenome at multiple levels. In the early 1980s, the gold standard technique developed for identifying differential DNA methylation took advantage of sodium bisulfite’s ability to selectively convert only unmethylated cytosine bases to uracil. After bisulfite conversion, both methylated and unmethylated DNA in regions of interest could be PCR amplified using flanking primers, cloned and Sanger sequenced [2]. Unfortunately, this technique carried a number of limitations, including the need for a significant fraction of an allele to be methylated, as well as significant technical expertise and resources for correct utilization [3]. Though bisulfite conversion and sequencing at individual loci allowed for many early discoveries in epigenetics, the significant labor and cost inherent to DNA sequencing led Herman and Baylin to later develop methylation-specific PCR (MSP) as a way to determine the methylation status of individual sites rapidly and efficiently. MSP utilizes primers specific only to methylated (or unmethylated) DNA, thus producing a PCR product after template bisulfite conversion only when bases of the correct methylation status are present [4]. The use of PCR as the step that distinguishes methylated vs. unmethylated DNA bases increases the sensitivity of the assay, allowing for measurement of significantly smaller amounts of DNA methylation than previous sequencing-based methods. More recently, by combining MSP with quantitative PCR and melting curve analysis, even more sensitive assays based on comparing ratios of methylated vs. unmethylated PCR products have been developed for measuring very low levels of methylation at specific loci [5]. Though MSP and its derivatives allowed for cataloging of methylation patterns in CpG islands of known tumor suppressors and oncogenes, with the increasing accessibility of genome sequencing, investigators have concomitantly become able to undertake epigenetic studies of broader scope. Whole genome bisulfite sequencing (WGBS) using massively parallel next generation sequencing technology has allowed for genome-wide examination of methylation patterns with single-base resolution. Nevertheless, a number of challenges continue to limit the utility of this technique. These include lower fragment complexity due to unmethylated cytosine to thymine conversion, PCR amplification bias for certain methylation states, significant coverage minimums, and difficulties with interpretation of methylation data in the context of other epigenetic changes, such as chromatin states [6, 7], as well as sequencing costs. As a result, multiple approaches have been developed to yield limited whole genome-level information while circumventing some of these limitations. Limited sequencing approaches attempt to reduce the cost and labor of WGBS by focusing on specific subsets within the larger genome. One widely used approach to genome-level methylation analysis is reduced representation bisulfite sequencing (RRBS). Rather than attempting to sequence the entire genome, RRBS begins with restriction enzymatic digestion of whole genomic DNA with a methylation-insensitive enzyme, frequently MspI, followed by size-based fragment purification. Fragments of between 40 and 220 base pairs have been shown to contain representative coverage and enrichment of most promoter and CpG island regions, which make up less than 1% of the genome [8, 9]. After bisulfite treatment, fragments are sequenced using traditional high-throughput sequencing methods. In addition to decreased cost and processing complexity, significantly less input DNA is required for RRBS vs. WGBS, allowing for RRBS use on paraffin-embedded or other limited tissue samples. RRBS also provides greater coverage in highly repetitive regions of the genome, including CpG islands, than WGBS, though RRBS ultimately only measures about 80% of CpG islands and 60% of promoters in the whole genome under optimal conditions [9]. For cancer genomics in particular, however, these advantages allow for the rapid comparison of large numbers of paired somatic and tumor epigenomes for a fraction of the price of whole genome sequencing. Recently, many additional methyl-sensitive endonucleases have been described, such as BisI, GlaI, and PcsI, which have different specificities and generate short fragments which can subsequently be sequenced [10]. Other limited sequencing approaches use microarray or other fixed probe-based capture techniques. BeadChip arrays, such as Illumina’s Infinium HumanMethylation450 system, have emerged as a widely used option for automated, low-cost, and rapid screening of pre-selected methylation sites for large numbers of samples [11]. Advantages include low starting material requirement (<1 μg DNA), relatively low cost, established analysis tools, and comprehensive promoter coverage. Some of the disadvantages include relatively high DNA purity requirement, lack of non-human genomes, and sparsity of non-promoter and non-CpG methylation probes [12]. Alternative approaches of focused differential methylation analysis include enriching for a subpopulation of DNA recognized by antibodies against 5-methylcytosine (MeDIP) or “pulling down” DNA using methyl-binding proteins (MBD-Seq) before sequencing. Comprehensive comparisons of currently available techniques and considerations regarding their use are available in literature [10, 12, 13].

## Aberrant methylation changes in melanoma

### Silencing of tumor suppressor genes

Focal DNA hypermethylation of tumor suppressor gene promoters is well described in many cancers, including melanoma. Silencing of PTEN, p16/14, and RASSF1A (Ras association domain family 1, isoform A) [14–19] in melanoma has been heavily investigated and is summarized in Table 1 [20]. The PTEN phosphatase converts PIP3 (phosphatidylinositol phosphate) to PIP2, antagonizing PI3K function, and suppressing activation of the PI3K/AKT pathway [21]. Mirmohammadsadegh et al. reported PTEN promoter methylation in 62% of melanoma serum samples examined [15]. Similar results were reported in a study using methylation-specific PCR, where 60% (120/200) exhibited methylation of the PTEN promoter [14]. PTEN methylation correlated with decreased immunohistological PTEN expression and was found to be associated with increased risk of death by Cox regression analysis. Recently, others have confirmed that methylation of PTEN is a significant prognostic factor of poor survival in melanoma [22]. In addition to epigenetic silencing, deletion/mutation occurs commonly and functional loss of PTEN occurs in approximately 40–60% of sporadic melanomas [23–25].

**Table 1:** Summary of well described tumor suppressor genes methylation in melanoma

Gene name	Function	Methylation prevalence (%)	Methylation context	Associated changes	Reference
PTEN	Inhibitor of PI3K signaling	6–62	Promoter hypermethylation	Gene transcription silenced	[14, 15, 22]
p16	Inhibitor of CDK4/6	5–27	Promoter hypermethylation	NRAS mutation associated	[16, 27, 29]
p14	Inhibitor of MDM2	41–57	Promoter hypermethylation	Gene transcription decreased	[17, 31]
RASSF1A	Cell cycle regulator	15–57	Promoter hypermethylation	Loss of expression	[19, 150]
MGMT	DNA repair	35	Promoter hypermethylation	No correlation	[150]

p16 is one of the proteins encoded by the CDKN2A locus and plays an important role in arresting the cell cycle at the G1/S checkpoint, by inhibiting CDK4 and CDK6 and activating Rb [26]. p16 promoter methylation was reported to occur in 25% of analyzed cutaneous melanoma metastases (15/59), with a significant overrepresentation in NRAS-mutated samples [16]. Additional studies reported similar frequencies of p16 methylation of 27% (16/60), albeit others have reported lower frequencies of approximately 5–10% [27–29].

p14, also encoded by the CDKN2A locus, binds and inhibits MDM2 from triggering p53 ubiquitination and targeting it for proteosomal degradation [30]. p14 methylation has been comparatively less studied but has been shown to be hypermethylated in approximately 57% (34/60 samples) of melanoma samples, independently of the p16 promoter (88% of the time), but also in conjunction with p16 promoter methylation (12% of the time) [31], findings that were later corroborated by additional studies [17]. Interestingly, CDKN2A methylation has not been reported in human melanocytic nevi (0/43 samples) [27, 32], in contrast to genetic alterations of this locus, which are well documented.

RASSF1A is a tumor suppressor gene, encoding a microtubule-associated protein that regulates mitotic arrest, cell cycle arrest, and apoptosis [33]. Methylation of two regions of the RASSF1A CpG island was reported in 44 metastatic melanoma tumors and 11 melanoma cell lines using methylation-specific PCR [19]. Overall, RASSF1A was hypermethylated in 55% of melanoma tumors, which correlated with loss of expression of the RASSF1A gene. The expression could be restored with 5-aza-2′-deoxycytidine treatment [19]. A follow-up study investigated the methylation of RARB2, MGMT, DAPK, and RASSF1A in 20 primary melanomas, 86 metastatic melanoma, and 15 cell lines [18]. RASSF1A was methylated in 15% (3/20) of primary tumors and 57% (49/86) of metastatic samples, suggesting that methylation level may correlate with advanced stage. Similarly, Tanemura et al. reported low methylation frequency of RASSF1A in early stage melanoma but approximately 50% in stage III and IV melanoma samples [34], suggesting that RASSF1A methylation may potentially be an indicator of tumor progression. Treatment with 5-aza-deoxycytidine causes demethylation of the RASSF1A promoter and re-expression [18]. A microarray-based screen identified 17 novel genes undergoing methylation-mediated silencing. Three genes have potential tumor-suppressive function, including HOXB13, SYK, and LXN [35, 36]. Methylation of genes classically thought of as oncogenes, such as KIT, has also been described in melanoma, with currently unclear functional roles [37].

### Methylation differences between melanocytes, nevi, and melanoma

Apart from known tumor suppressors, over a hundred other genes have been identified as differentially methylated in melanoma relative to melanocytes. Many differentially methylated genes belong to pathways critical for cancer cell survival and growth, including cell cycle, apoptosis, metabolism, DNA repair, PI3K/mTOR signaling, metastasis, and immune response, as summarized in Table 2. Many genome-wide studies have investigated the methylome of melanoma cell lines and clinical samples [35, 38–41]. The first genome-wide integrated analysis of promoter methylation and gene expression compared methylation and expression in eight melanoma cell lines relative to newborn and adult benign melanocytes [39]. The study used an elegant pipeline of linear mixed-effect modeling and manual promoter region filtering. It identified 76 differentially methylated markers, most of which—89% (68/76), were hypermethylated, and only a minority was previously reported in melanoma at the time (COL1A2, RAB33A, DDIT4L, and HOXB13). The results were validated by bisulfite sequencing of COL1A2, NPM2, HSPB6, DDIT4L, and MT1G promoters, which exhibited increasing incidence with advanced melanoma stage, suggesting potential use as predictors of melanoma progression [39]. A subsequent study used a candidate gene approach and investigated the methylation status of 15 cancer-related genes in 16 melanoma cell lines. Melanoma hypermethylation prevalence for ERα (50%), MGMT (50%), RARB2 (44%), RIL (88%), RASSF1A (69%), PAX7 (31%), PGRB (56%), PAX2 (38%), NKX2-3 (63%), OLIG2 (63%), HAND1 (63%), ECAD (88%), CDH13 (44%), MLH1 (0%), and p16 (6%) was reported [40]. Similarly, Bonazzi et al. reported methylation of COL1A2 (24%), THBS1 (31%), TNFRSD10D (66%), and UCHL1 (42%) genes in 12 melanoma cell lines, relative to a reference pool of melanocytes [42]. More recent studies have integrated DNA methylation changes with transcriptional changes (RNA-Seq) and histone modifications (ChIP-Seq). An integrated RNA-Seq and CpG island demethylation profiling of melanocytes and melanomas found that melanocytes undergo progressive hypomethylation, followed by extensive hypermethylation as melanoma progresses [43]. However, these results are based on one stage I melanoma cell line and melanocyte line, one stage III melanoma cell line, and two stage IV melanoma cell lines. Despite these limitations, the study was able to correctly ascertain the methylation status of PCSK1, CYP1B1, QPCT, c-Kit, and TERC genes, which were previously identified by candidate gene approaches. Overall, 821 differentially methylated genes were identified, mostly belonging to a network of developmental pathways [43]. Notably, repetitive DNA elements exhibited extensive hypomethylation in stage III/IV melanoma cell lines, possibly contributing to increased transposition and mutagenesis [43]. The study provided a comprehensive catalog of CpG methylation and expression during melanoma progression, which could be utilized as a resource to better understand how methylation changes evolve in melanoma.

**Table 2:** Pathway classification of differentially methylated genes in melanoma

Pathway/function	Genes (methylation frequency^a^)	References
Tumor suppressors	CDH1 (88%), CDKN2A (76%), PTEN (23%), APC (15%), SOCS1 (75%)	[15, 40, 151, 152]
Oncogenes	PAX7 (31%), OLIG2 (63%), SYK (2%)	[35, 40]
Protein kinases	DAPK1 (19%), HSPB8 (69%)	[152, 153]
Differentiation	TNFRSF10A/C/D,CCR7, THBD (20%), BST2 (50%), DPP4 (80%), ENC1 (6%), GDF15 (75%), WIF1	[35, 154–159]
Homeodomain proteins	HOXB13 (20%), PAX2 (38%), PAX7 (31%), NKX2-3 (63%)	[35, 40, 160]
Hypoxia	CDKN1B, CDKN1C (35%), CXCR4, LXN (95%)	[35, 36, 151, 154]
Transcription factors	PGR (56%), HAND1/2 (15%), PAX7 (31%), ESR1, RUNX3 (23%)	[40, 151, 161]
Interferon gamma response	SOCS1 (75%), HLA-A, PTGS2 (20%), SOCS3 (60%), BST2 (50%), XAF1, SOCS2 (44%)	[35, 152, 162–164]
Epithelial-to-mesenchymal transition	TFPI2, TIMP3 (13%), COL1A2 (63%), TPM1 (8%), PDLIM4	[39, 42, 152, 165, 166]
PI3K/mTOR	CXCR4, CDKN1B, PTEN (23%)	[151, 154]
Metastasis	CDH8 (10%), CDH13 (44%), EPB41L3 (5%), SERPINB5 (100%), TFPI2, SYK (3%), CCR7	[35, 40, 152, 154, 155]
Immune recognition	MAGE-A1,2,3,4, BAGE (83%), HLA-A	[167, 168]
Apoptosis	RASSF1A, HSPB6 (69%), TRAILR1 (80%)	[39, 169, 170]
DNA repair	MGMT (0–50%)	[151]
Metabolism	CYP1B1 (100%), DNAJC15 (50%), CD98	[35, 43, 171]
Other/unknown	FAM78A, LRRC2, PCSK1, PPP1R3C, PTPRG, QPCT, SLC27A3, DERL3, MFAP2, MT1G, WFDC1	[35, 39, 42, 151]

^a^Where applicable

Significant methylation differences have also been reported between melanocytic nevi and melanoma. A study using microarrays to survey 1505 CpG sites in 27 benign nevi and 22 primary melanomas identified significantly differentially methylated genes that distinguished melanoma from nevi [38]. Seven genes were reported as hypermethylated in melanoma (COL1A2, FRZB, GSTM2, KCNK4, NPR2, TRIP6) and 19 genes were reported as hypomethylated, (CD2, EMR3, CARD15, EV12A, HLA-DP1, IFNG, IL2, ITK, KLK10, LAT, MPO, PSCA, PTHLH, PTHR1, RUNX3, and TNFSF8). Approximately half of the genes had immune-related functions, including T cell regulation (ITK, LAT, CD2, TNFSF8, IFNG, and IL2). However, the study did not differentiate between expression in melanoma lesional cells or infiltrating lymphocytes [38]. Another group studied global DNA methylation in melanoma cell lines and benign nevi using Infinium BeadChip arrays and identified 106 genes hypermethylated in melanoma [41]. The most frequently hypermethylated genes identified were HOXA9, C1orf106, HIST1H3E, MAPK13, and LEP, with MAPK13 methylation in 67% of primary and 85% of metastatic melanomas. Interestingly, transfection of ectopic MAPK13 into cell lines with promoter methylation of MAPK13 decreased proliferation, but did not affect proliferation of cells without endogenous MAPK13 silencing. Gene set enrichment revealed significant overrepresentation of homeobox genes (HOX) and G protein-coupled receptor genes [41]. While numerous DNA methylation differences were identified among melanocytes, nevi, and melanoma, no methylation-based clinically validated assays have been reported to date.

### Methylation clusters in melanoma are associated with different biologic behavior

Several studies have suggested that distinct methylation subgroups exist within melanoma and are associated with distinct biologic behavior and survival outcomes. Unsupervised clustering and principal component analysis of genome-wide DNA methylation patterns in melanoma revealed three methylation clusters based on total methylation: MS1, MS2, and MS3 [44]. The MS1 group had the highest methylation level, mainly elevated at promoter islands and poised (“bivalent”) promoters, which are Polycomb repressive complex (PRC2) target genes. The MS3 group had the lowest methylation levels, resembling peripheral blood leukocytes, and MS2 was intermediate. Neither BRAF nor NRAS mutation was clearly associated with the methylation clusters, but homozygous deletions of CDKN2A were more frequent in MS1. There was no significant association of the methylation clusters with histopathology or primary tumor characteristics. Furthermore, the subgroups did not differ by the total number of mutations, but MS1 was enriched for IDH1 R132 hotspot mutations. Metastatic melanoma samples from TCGA with the MS1 signature had significantly inferior survival (~20 months for MS1 vs. 60 months for MS3) [44]. Analyzing differential gene expression between the clusters revealed upregulation of TP53, MDM2, CDK4, CDK6, CCND1, CCNE1, and E2F3, as well as epigenetic modifiers TET1, JARID1B, SWI/SNF chromatin remodelers (SMARCA1/2/4, ARID2, PBRM1), and DNMT3A in the MS1 cluster, and enrichment of an immune signature in the MS3 cluster. The clusters were correlated with different phenotypic behavior based on gene expression analysis. The MS1 cluster was termed “proliferative,” while the MS3 cluster was dominated by an “immune high” signature, possibly explaining the better survival of MS3 cluster patients [44]. However, it remains unclear which immune cells infiltrate and how they interact with melanoma cells. In contrast to these findings, Ecsedi et al. suggest that hypermethylation is an early event in melanoma, that is, methylation gradually decreases with progression (inversely correlated with Breslow thickness), and is associated with less favorable overall survival [45]. They also found that BRAF V600E is associated with specific methylation changes, as opposed to wild-type samples. This finding is consistent with studies suggesting that oncogenic BRAF drives methylation of specific target genes [46, 47]. It should be noted that Ecsedi et al. used an assay method that surveys only 1505 CpGs, as opposed to Lauss et al. who used the HumanMethylation450K platform (~480,000 CpGs). The different findings could possibly be explained by differences in metastatic and primary tumors, use of different methodologies with different scope (Methyl450 vs. GoldenGate Methylation assay), or underlying differences in the sampled populations. Both studies support the existence of at least two distinct methylation clusters in melanoma, which are associated with different gene expression and prognosis. Others have reported genome-wide methylation correlating with Breslow thickness, mutated BRAF, ulceration, and a negative association with mitotic rate [48]. A potential role for DNA methylation in regulating metastasis to the brain was linked to aberrant methylation of the homeobox D cluster [49]. Identifying differentially methylated CpG islands during melanoma progression suggested that genome-wide overall DNA methylation (mostly intragenic) significantly decreased with progression but focal (promoter associated) CpG methylation progressively increased. Analyzing methylation differences between brain metastases and lymph node metastases identified ~1500 differentially methylated CpG sites associated with genes. Enrichment analysis revealed overrepresentation of genes involved in cell cycle regulation, morphology, and assembly, and DNA repair, recombination, and replication [49]. Integrating methylation changes with gene expression changes prompted focus on the homeobox (HOX) gene family, with 10 members of the family undergoing differential methylation [49]. Specifically, HOXD9 was hypermethylated in brain metastases and associated with a 2.7-fold higher risk of death. HOX genes play important roles during development but are silenced in somatic cells by Polycomb repressive complexes. Aberrant expression of HOX genes was reported in different human tumors, including melanoma [50]. SOX9 expression has also been shown to be regulated by DNA methylation in melanoma, where it can regulate invasion, and predict poor survival [51]. In addition to observational studies describing the methylome of melanoma, several studies suggest a BRAF-driven mechanism of aberrant methylation of specific genomic loci in melanoma [46].

### DNA methylation of miRNA genes in melanoma

**Fig. 1:**
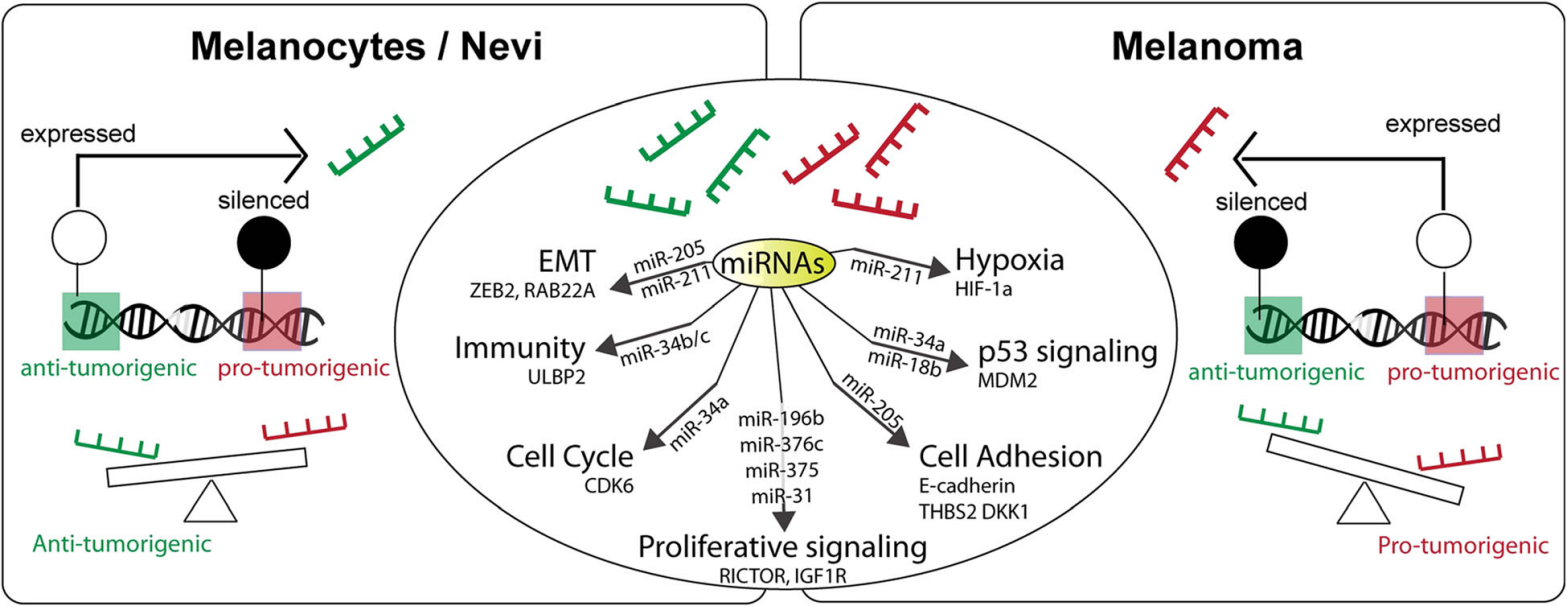
Abnormal DNA methylation in melanoma disrupts microRNA expression. In melanocytes and nevi (*left*), normal DNA methylation patterns contribute to balanced pro-tumorigenic (*shown in red*) and antitumorigenic (*shown in green*) microRNA expression. In melanoma (*right*), aberrant DNA methylation causes expression changes leading to microRNA imbalance and abundance of pro-tumorigenic microRNAs. See text for specific microRNAs disrupted and their targets

Methylation of microRNA (miRNAs) genes has also been shown to play important roles in melanoma cell survival, proliferation, and migration by affecting epithelial-to-mesenchymal transition (EMT) signaling, cytoskeletal components, p53, and PI3K pathways (Fig. 1). miR-34a methylation and expression silencing was reported in 43% (19/44) of melanoma cell lines and 60% (20/32) of primary melanomas [52]. miR-34a, which is also a p53 transcriptional target, is thought to play a tumor-suppressive role in cancer by inducing cell cycle arrest or apoptosis, at least partly by regulating CDK6 [52]. A liposomal miR-34a mimic recently showed potential activity in acral melanoma in a phase I clinical trial [53]; however, the expansion study (NCT01829971) was subsequently halted due to multiple grade 4 immune-related adverse events. Other members of the miR-34 family, miR-34b and miR-34c, were reported to regulate the expression of natural killer cell receptor ligand ULBP2 [54], a prognostic marker in human melanoma [55], as well as MET, cyclin-dependent kinases, and N-myc expression [56, 57]. Forced expression of miR-34b decreases melanoma cell invasion and adhesion, presumably through affecting cytoskeletal remodeling and expression of cell adhesion network genes, such as THBS2 and DKK1 [58]. miR-211 expression is regulated by DNA methylation (DNMT1) in melanoma and can act as a metabolic switch through regulating HIF-1a protein stability [59] and inhibiting EMT by targeting RAB22A [60]. Ectopic expression of miR-211 in melanoma is associated with decreased cell growth during hypoxia [59]. DNA methylation is also thought to regulate the expression of miR-203 [61]. miR-203 ectopic expression was reported to decrease cell invasion by targeting the Polycomb group gene BMI1 in melanoma [62]. Additionally, Mazar et al. reported that miR-34b/c, miR-489, miR-375, miR-132, miR-519b, miR-654, miR-let-7e, miR-142-3p, miR-200a, miR-145, miR-21, miR-496, and miR-452 expressions can be induced by hypomethylating agents in melanoma cell lines [58, 63]. miR-375-associated CpG island methylation and suppression was reported in melanoma tissue samples from primary and metastatic lesions, but not in melanocytes or melanocytic nevi. Expression of miR-375 could be de-repressed with hypomethylating agents, and ectopic expression inhibited cell proliferation, invasion, and motility [64]. Expression of miR-18b also was reported to be silenced by DNA methylation of a proximal CpG island in melanoma cell lines. Forced re-expression decreased MDM2 and increased p53 expression level, associated with suppression of colony formation [65], suggesting a tumor-suppressive role for miR-18b. Methylation and silencing of miR-196b expression was reported to potentiate mTOR signaling in melanoma [66] and other cancers [67]. miR-205 was reported to be silenced in melanoma compared to melanocytic nevi. Ectopic expression of miR-205 decreased melanoma cell migration in vitro, associated with increased decreased expression of ZEB2 and increased expression of E-cadherin [68]. Silencing of a large miRNA cluster on chromosome 14q32 is reported to be mediated by DNA methylation in melanoma. Within this cluster are miR-376a and miR-376c, which can downregulate IGF1R levels and decrease cell migration and growth [69]. miR-31 is also frequently silenced by DNA methylation in melanoma cells, and forced expression of miR-31 is reported to decrease cell migration and invasion, possibly through targeting SRC, MET, RAB27a, and MAP3K14 [70].

Most studies described above suggest that hypermethylation and silencing of miRNAs plays a pro-tumorigenic role in melanoma and that ectopic expression of specific miRNAs could attenuate melanoma proliferation and metastatic potential by targeting EMT signaling, PI3K/AKT pathway, or p53 signaling in vitro and in animal models. Despite solid pre-clinical evidence, very few miRNA liposomal therapies have been investigated in clinical trials in melanoma thus far.

### Source of aberrant DNA methylation

The source of aberrant DNA methylation in melanoma, i.e., global hypomethylation and focal hypermethylation, remains elusive [22]. In general, these changes could be mediated by (1) an active process, either through increased activity or overexpression of DNMT enzymes; (2) aberrant targeting of DNMT enzymes, due to altered splicing, altered binding, or altered expression of scaffolding proteins; and (3) a passive process due to changes in other epigenetic modifications that regulate targeting of DNA methylation. The notion that DNA methylation is regulated by, and regulates, proliferative signaling will be discussed here, due to emerging evidence supporting this hypothesis.

PI3K signaling, which is activated in most cancers, can regulate methylation of imprinted regions [71], and inactivation of PI3K signaling decreases DNMT3B level in HCC [72], melanoma, and prostate [73] and other cancers. Aberrant DNA methylation may thus act as a licensor that enables chronic activation of growth-signaling pathways, i.e., PI3K/mTOR signaling through various mechanisms, which are probably cell type specific, but may include PTEN silencing, miRNA-mediated regulation of RICTOR [66], or other components of the PI3K/AKT pathway. Transient activation of proliferative pathways may serve to instruct proliferative DNA methylation patterns by upregulating DNMT expression, altering DNMT splicing, or altering DNMT targeting, by affecting expression of targeting proteins (such as MAFG) [47]. Once aberrantly activated, misguided DNA methylation leads to establishment of abnormal patterns that support chronic activation of growth signaling. BRAF activation and MAPK signaling has been shown to drive tumor-specific methylation programs in colon cancer and possibly melanoma [47]. Our in vivo reports in melanoma and others in specific CNS tumor support [74, 75] this notion, but it remains to be thoroughly investigated in vivo in additional cancers. In vitro, many cancers require DNMT3B for survival and proliferation [76], implying that DNMT3B can regulate proliferative pathways in a wide array of cancers. Apart from this instructive (“driver”) role targeting particular pathways, it is also plausible, but less likely, that aberrant DNA methylation leads to stochastic expression changes that promote tumor progression. CpG microarray analysis of UACC62 melanoma cell lines with and without BRAF knockdown surprisingly identified many hypermethylated and hypomethylated genes, suggesting that mutant BRAF dictates a specific methylation program. Knockdown of BRAF decreased the expression of DNMT1 and EZH2 in the cell line, suggesting that DNMT1 may be the mediating DNMT. However, expression of other DNMTs was not investigated, and rescue of demethylated sites by DNMT1 overexpression was not reported. In addition to proliferative pathways regulating DNA methylation, the converse is also described, establishing a potential possible feedback loop: methylation has been shown to regulate MITF expression, SOX9 expression, MAPK, and PI3K signaling [51, 77, 78]. Recently, loss of LKB1, a tumor suppressor somatically inactivated in ~10% of melanomas, has been shown to funnel glucose derivatives toward serine metabolism, increased S-adenosyl methionine production, DNA hypermethylation, and increased proliferation in the context of Ras/Raf activation [79].

### DNA demethylation in melanoma

Shortly following the discovery of 5-hydroxymethylcytosine (5-hmc), active demethylation, and the role of TET family hydroxylases in converting 5-mc to 5-hmc [80–82], an important role for 5-hmc was shown in melanoma [83]. Lian et al. investigated the global level of 5-hmc in human melanocytes, nevi, and melanoma and reported a significant decrease of 5-hmc in melanoma relative to nevi and melanocytes. The loss of 5-hmc correlated with melanoma progression in patient sample tissue microarrays. Importantly, overexpressing IDH2 or TET2 restored 5-hmc levels in melanoma cell lines, suggesting that lack/loss of function of these enzymes plays an essential role in 5-hmc depletion during melanoma progression. Restoring 5-hmc levels by IDH2 and TET2 overexpression was associated with suppressed tumor invasion and growth in a zebrafish melanoma model and mouse xenograft model of melanoma [83], suggesting a potential functional role of this epigenetic mark in melanoma growth and invasion. Several subsequent studies confirmed that 5-hmc is decreased in metastatic melanoma, associated with loss of TET activity [84, 85], and suggest its use as an adjunct marker to distinguish between benign nodal nevi and melanoma in lymph node biopsies [86–88]. While conclusive functional studies in melanoma have not been reported yet, 5-hmc may be enriched in exons and transcription factor binding sites to promote gene expression [89, 90], through distinct 5-hmc readers and transcription factors [91, 92], according to studies in stem cells and CNS. Mutations in IDH1/2 enzymes have been reported in ~6–10% of melanomas [93, 94] and correlate with the CpG island methylator phenotype (CIMP) cluster-defined DNA methylation profile in melanoma samples in TCGA. TET enzyme family expression has been reported as decreased in melanoma, albeit by an unknown mechanism [83, 85]. Certain IDH1 mutations result in gain of function production of oncometabolite 2-HG, a competitive inhibitor of α-KG, which is a necessary cofactor for TET enzymatic activity. An SNP associated with melanoma risk was discovered in an intron of TET2, and somatic mutations of TET2 were reported in approximately 4% of melanoma cases in one study [95]; however, other sequencing studies have not reported TET mutations [93, 96].

Our current understanding of the role of DNA demethylation and oxidative derivatives of 5-mc in melanoma is in its infancy. Currently, there is a large discrepancy between the frequency of loss of 5-hmc in melanoma (nearly universal, according to several reports) and mechanisms potentially explaining loss of 5-hmc (IDH gain of function, TET enzyme loss of function). Additionally, studies investigating 5-fc (5-formylcytosine) and 5-cac (5-carboxycytosine) prevalence and distribution in melanoma have not been reported yet. Early evidence suggests that DNA demethylation plays important roles in melanoma biology and will undoubtedly be a field of active investigation in years to come.

## Differential DNA methylation as a biomarker in melanoma

DNA methylation has the potential to be used as a melanoma biomarker in several clinically important settings: (1) differentiating benign lesions (nevi) from melanoma; (2) determining melanoma burden in sentinel lymph nodes; (3) aiding prognosis of stage III patients, which currently exhibit great variability in survival; (4) predicting response to therapies; and (5) monitoring for recurrence using peripheral blood.

Conway et al. identified a 12 CpG locus signature highly predictive of melanoma in a small independent sample set of 29 nevi and 25 melanomas (ROC = 0.95) [38]. Gao et al. reported a diagnostic algorithm consisting of CLDN11, CDH11, PPP1R3C, MAPL13, and GNMT which could help distinguish between melanoma and dysplastic nevi (ROC = 0.81) [97]. Detection of aberrantly methylated SOCS1/2, RASSF1A, MGMT, and CDKN2A in serum has been reported to have diagnostic value in patients with malignant melanoma [98]. Despite reasonable success with the small validation cohorts in the original reports, these signatures have not been further validated by other, larger studies.

Melanoma patients currently exhibit great variability in survival, even within AJCC subgroups [99], and there is great unmet need for a molecular biomarker to further stratify patient risk. Identification of a CpG island methylation signature has been useful in predicting prognosis, diagnosis, and response to treatment in a variety of tumor types [100, 101]. Genome-wide methylation studies have uncovered that tumors with enriched methylation in CpG islands have a particularly aggressive phenotype, termed the CpG island methylator phenotype (CIMP) [102]. CIMP was found to predict prognosis and, in some cases, response to therapy in various cancers [103–109]. Tanemura et al. investigated promoter methylation of tumor-related genes (TRGs): WIF1, TFPI2, RASSF1A, SOCS1, GATA4, RARB2, and a family of MINT (methylated in tumor) genes in an attempt to define a CIMP in melanoma [34]. They reported an association of WIF1, TFPI2, RASSF1A, and SOCS1 hypermethylation with advanced clinical stage in a small cohort of patients (*n* = 122). Apart from this proposed CIMP signature, several studies reported that DNA methylation of single genes can predict survival of melanoma patients. Lahtz et al. reported that methylation silencing of PTEN, an inhibitor of PI3K signaling, exhibited significant association with poorer survival in melanoma [14].

Sigalotti et al. reported that methylation level of LINE-1 is associated with shorter overall survival in stage III cutaneous melanoma [110]. The 5-year overall survival with hypomethylated LINE-1 was 48%, compared to 7% for hypermethylated sequences [110]. In contrast, Ecsedi et al. reported that hypomethylation of LINE-1 is associated with shorter survival [111] and additionally identified hypermethylation of six genes (DSP, EPHB6, HCK, IL18, IRAK3, and KIT) as a poor prognostic factor associated with decreased overall survival. You et al. reported that increased TSLC1 (tumor suppressor in lung cancer 1) promoter methylation is associated with advanced melanoma stage and shorter progression-free survival in a cohort of 120 melanoma samples [112].

Methylation of the DNA repair protein MGMT was reported to predict response to temozolomide treatment in stage IV melanoma patients [113]. Of 64 patient samples in the study, methylation of MGMT was detected in 25% (16/64) of the samples. In the hypermethylated group, the response rate to temozolomide was 62%, compared to 15% in the unmethylated group. MGMT promoter hypermethylation was also associated with significantly higher response rate and longer progression-free survival in other studies [113, 114]. It should be noted that MGMT methylation status predicts response to temozolomide in glioblastoma [115, 116] and is used clinically for identifying tumors that may respond to temozolomide. Presence of circulating methylated RASSF1A DNA in the blood of melanoma patients was reported as a predictor of therapy response and disease outcome [117]. Circulating methylated RASSF1A DNA was associated with lack of response to therapy, while detection of at least one methylated gene out of RASSF1A, RAR-beta2, and MGMT signatures was associated with shorter overall survival [117]. A study investigating differentially methylated CpG sites between melanoma brain metastases (poor prognosis) and lymph node metastasis from patients with good prognosis reported that HOXD9 methylation was associated with overall survival [49]. In a validation cohort of 145 melanoma patients, HOXD9 hypermethylation in lymph node metastases was associated with shorter progression-free and overall survival [49]. The function of HOXD9 in melanoma is incompletely understood, but studies from HCC suggest that it significantly enhances migration, invasion, and metastasis [118]. Methylation and silencing of ESRP1 has been also associated with altered CD44v6 expression, which could predict brain metastasis-free survival [119].

Sigalotti et al. used genome-wide Methylation 27 beadchip arrays to study 45 stage IIIc melanoma patients [120]. Using *K*-means clustering, the cohort was classified into a favorable and an unfavorable group, based on global methylation. Median survival in the favorable group was 31.5 months, compared to 10.3 months in the unfavorable group, translating to 5-year overall survival of 41.2 and 0%, respectively [120]. Samples could also be correctly assigned to groups using a 17-gene nearest shrunken centroid methylation signature (FGF4, WNT10B, FLJ33860, PCDHAC2, SLC6A11, GJB5, S100A9, CRHR1, MGC35206, IGLL1, TRIM40, SLC18A2, TUB, GRM4, SLC6A18, and ALOX12B). Methylation of the miR-196b promoter has been associated with a poorer 5-year overall survival in melanoma patients in TCGA by Cox multivariate analysis ([66, 93]).

Bell et al. studied enhancer methylation in a panel of 23 cancers, including melanoma. They reported that differentially methylated enhancer regions were metastatic site specific and determined by microenviromental cues. A significant correlation was found between specific enhancer methylation patterns and patient survival. Specifically, differential methylation of KIT-, CYTL1-, and KIF14-associated enhancers played a role in regulating proliferation [121].

While important and informative, the main limitation of the above studies is relatively small sample cohorts, lack of subsequent validation studies, and reliance on melanoma cell lines (in some studies), which can have an altered DNA methylation landscape. With the availability of large, well-annotated melanoma sample databases, such as TCGA and the International Cancer Genome Consortium, it will now be important to validate the status of the biomarkers discussed above and develop the most promising markers with prospective studies into a methylation signature which can be of clinical utility. Studies investigating DNA methylation as a diagnostic and prognostic tool in melanoma are summarized in Table 3.

**Table 3:** Summary of DNA methylation biomarkers in melanoma

Methylated gene(s)	Methylation association	Hazard ratio^a^	Reporting study
WIF1, TFPI2, RASSF1A and SOCS1	Advanced clinical stage	–	[34]
17-gene (see text)	Overall survival	2.41 (1.02–5.7)	[120]
RASSF1A, RAR-beta2, MGMT	Shorter overall survival	2.38 (1.1–4.8)	[117]
KIT, DSP, HCK, IL18	Shorter overall survival	–	[45]
HOXD9	Shorter overall survival	2.7 (1.1–6.5)	[49]
LINE-1	Shorter overall survival	2.63 (1.2–5.6)	[110]
MINT31	Longer overall survival	0.237 (not reported)	[34]
TFPI2	Presence of metastases	–	[172]
PTEN	Shorter overall survival	3.76 (1.2–11.1)	[14, 22]
MGMT	Response to therapy	-	[113]
	Longer progression-free survival	2.17 (1.3–3.5)	
TSLC1	Progression-free survival	-	[112]
RASSF1A	Resistance to therapy	0.21 (0.1–0.9)	[117]
ESR1	Shorter overall survival	2.31 (1.4–5.5)	[161]
	Shorter progression free survival	2.64 (1.3–5.1)	
p73	Sensitivity to therapy	–	[173]

^a^Where applicable, with 95% confidence interval in parenthesis

## DNA methylation as a therapeutic target in melanoma

### Nonspecific inhibition of DNA methylation

The reversible nature of DNA methylation makes it a desirable drug target, as opposed to mutations and deletions, which are more challenging to rectify [122]. Two major demethylating agents, 5-aza-2-deoxycytidine (decitabine, DEC) and 5-azacitidine (azacitidine, AZA), are FDA approved for treating myelodysplastic syndrome (MDS) and certain cases of AML [123]. It is still debated whether the clinical efficacy of high-dose AZA is due to demethylating activity [124] or DNA methylation-independent roles [125], notably induction of cytotoxic DNA damage, cell cycle arrest, and induction of tumor suppressors. FDA-approved use of demethylating agents currently remains limited to few hematologic malignancies. The role of decitabine in hematologic malignancies is well recognized with ~75 open clinical trials registered (most for myelodysplastic syndrome and AML). However, there are comparatively few active trials in solid tumors. A phase I/II ongoing clinical trial (NCT01876641) is investigating decitabine in combination with vemurafenib as a treatment for treatment-resistant BRAF V600-mutated metastatic melanoma. Preliminary results, presented at the ASCO 2015 annual meeting, suggest a 21% complete response rate. Complete response rates of <1% seen with vemurafenib alone were reported in the BRIM-3 trial [126], suggesting that DNA methylation may possibly play a role in regulating MAPK signaling and/or resistance to vemurafenib (J Clin Oncol 33, 2015 suppl; abstr 9056). Based on recent results, targeting DNA methylation may also be a synergistic strategy in those melanomas harboring somatic inactivation of the LKB1 tumor suppressor [79].

### Inhibition of individual DNMT enzymes

**Fig. 2:**
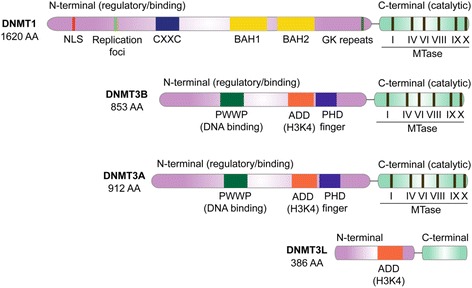
Structural domains of mammalian DNA methyltransferases. *NLS* nuclear localization signal, *BAH* bromo-adjacent homology, *GK* Gly-Lys linker, *CXXC* cysteine-rich motif, *MTase* methyltransferase, *PWWP* Pro-Trp-Trp-Pro motif, *ADD* ATRX/DNMT3/DNMT3L, *PHD* plant homeodomain

DNA methylation in mammals is established and maintained by the complex interplay of at least three enzymatically active, independently encoded DNA methyltransferases: DNMT1, DNMT3A, and DNMT3B [127–129], summarized in Fig. 2. An additional DNMT3-like protein, DNMT3L, lacks essential components of the MTase (methyltransferase) motif but can bind and regulate the activity of DNMT3A [130] and DNMT3B [131, 132]. The non-redundant roles and specific targeting preferences of DNMTs are just becoming appreciated, owing to elegant enzyme-specific disruption studies [133, 134]. Consistent with this idea, a division of labor among methyltransferases [133] has been proposed and DNMT3B in particular may play specific, non-redundant roles in gene body methylation [135]. While there are currently no DNMT-specific inhibitors, there is extensive evidence suggesting that inhibition of DNMTs may have an antiproliferative effect on melanoma cells.

Antisense depletion of DNMT1 in the MZ2-MEL cell lines was shown to lead to hypomethylation and re-expression of the germ line-specific MAGE-A1 transgene, which is commonly silenced in melanoma [136]. Cancer-germ line gene activation upon DNMT1 transient depletion was observed in 45 human melanoma cell lines using a microarray approach [137]. Ras/Rac1/ERK activation induced increased DNMT1 protein expression during anchorage blockade and in response to oxidative stress. Interestingly, PI3K/AKT activity did not affect DNMT1 protein levels [138]. This study further proposed an increase in DNMT1 as a mediator of global DNA methylation changes and contributor to malignant transformation, although these claims have not been functionally investigated.

Stable RNAi depletion of DNMT3A in B16 melanoma cells decreased tumor growth and metastasis in a xenograft mouse model [139], which was associated with dysregulation of class I and class II MHC genes. The authors hypothesized that loss of DNMT3A increased MHC-dependent antigen presentation, prevented immune escape, and led to a T cell-mediated immune response, but this remains to be functionally investigated.

DNMT3B expression increases with melanoma progression [140], and DNMT3B has been associated with p16INK4A methylation in melanoma [17], as well as regulating mTORC2 signaling in a melanoma model in vivo [66]. Despite many pre-clinical studies suggesting a pro-tumorigenic role for individual DNMT enzymes, there are currently no enzyme-specific inhibitors. This is partly due to incomplete understanding of allosteric control, lack of crystal structures, and complex interactions, i.e., heteropolymer formation of DNMT enzymes [141].

### DNA methylation and immunotherapy

**Fig. 3:**
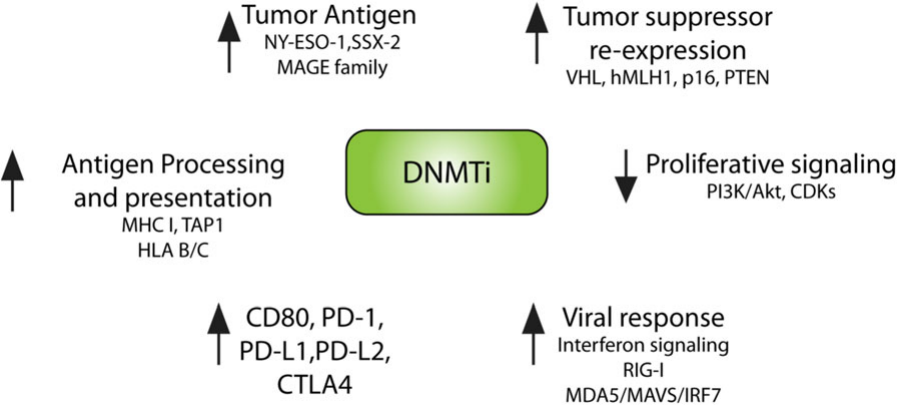
Potential mechanisms of synergy between DNA methylation inhibitors with targeted therapies and immune-checkpoint therapy. Inhibition of DNMTs has been shown to increase expression of melanoma antigens such as NY-ESO-1, MAGE family genes, and SSX-2. Inhibition of methylation can lead to tumor suppressor (VHL, hMLH1, p16, PTEN) re-expression, decreased proliferative signaling, increased viral response through activation of interferon signaling, altered expression of immunocheckpoint receptors, and increased antigen processing

The most promising novel therapy for melanoma, and other cancers with many neoantigens such as NSCLC, is immune-checkpoint inhibition [142]. DNA methylation has been long recognized to regulate expression of antigen presentation genes, MHC class I genes, tumor antigens such as MAGE and NY-ESO1, viral response, and interferon pathway genes [143]. DNA methylation was also recently shown to regulate expression of PD-1, PD-L1, PD-L2, and CTLA-4 [144]. Furthermore, many known methylation targets have been identified as differentially expressed in response to anti-CTLA-4, anti-PD-1 combination, or sequential therapy [145]. Perturbing DNA methylation could thus be a potential pathway to augment antitumor immune responses, and pre-clinical evidence supporting this notion has recently emerged. Inhibition of DNA methylation using AZA was shown to increase effectiveness of anti-CTLA4 therapy in a B16 graft model of melanoma in vivo [146] and reactivate expression of endogenous viruses in colon cancer cell lines [147]. Others have identified a distinct melanoma methylation cluster with improved survival rates and overexpression of an immune signature [44]. However, it is difficult to delineate whether the increased sensitivity to anti-CTLA4 is the result of specifically inhibiting DNA methylation and tumor antigen re-expression, triggering interferon signaling, DNA damage, DNA methylation-independent antiproliferative effects of AZA, or some other mechanism. Clinically, lung cancer patients who received AZA were subsequently more likely to respond to immune-checkpoint inhibitors [148]. Sporadic microsatellite instable colorectal cancer, which is associated with promoter methylation of Wnt target ITF2 and hMLH1, has been shown to respond to PD-1 inhibitor immunotherapy in a small trial [149]. Clinical trials combining 5-azacitidine with nivolumab and ipilimumab are currently ongoing in MDS, AML, and NSCLC. In melanoma, a phase I clinical trial (NCT02608437) is currently evaluating combination treatment with guadecitabine (hypomethylating agent) and ipilimumab in patients with unresectable or metastatic disease (J Clin Oncol 34, 2016 suppl; abstr TPS9595). Similarly, combination of oral azacitidine with pembrolizumab is being evaluated as part of a phase II trial in metastatic melanoma (NCT02816021). Results of these clinical trials (Table 4) will provide important information regarding the potential synergistic effects of hypomethylating agents and checkpoint inhibitors in melanoma (Fig. 3).

**Table 4:** Summary of current clinical trials targeting DNA methylation in melanoma

Approach	Phase	Status	Clinical trial identifier
Guadecitabine + ipilimumab for unresectable disease	I	Recruiting	NCT02608437
Oral azacitidine + pembrolizumab for metastatic melanoma	II	Recruiting soon	NCT02816021
Azacitidine + rInterferon alfa2b for stage III/IV unresectable	I/II	Completed, results not reported	NCT00217542
Oral azacitidine bioequivalence study	I	Recruiting	NCT02223052
Decitabine + vemurafenib + cobimetinib for resistant disease	I/II	Recruiting	NCT01876641

## Conclusions

Identification and cataloging of aberrant DNA methylation changes in melanoma, as reported by numerous studies discussed above, is an important first step toward understanding the methylation landscape of human melanoma and utilizing it for clinical benefit. Methylation of immunomodulatory genes, existence of distinct DNA methylation clusters with separate molecular phenotypes, and aberrant methylation of Polycomb targets are some of the general trends emerging in recent literature. However, many challenges remain to be resolved before the potential of targeting DNA methylation can be optimally utilized. Functional evaluation of differentially methylated genes in vivo, i.e., whether the aberrant methylation is directly driving proliferation or is a by-product of malfunctioned methylation machinery (“passenger”), is generally lacking. The origin of aberrant DNA methylation, i.e., altered expression and/or targeting of DNMT enzymes, remains incompletely understood, and identification of DNMT-specific inhibitors has proven to be challenging. Finally, understanding the molecular correlates of melanoma response to immunotherapy is a tremendously important question and one in which DNA methylation may play a crucial role, according to recent studies. Clinical trials investigating potential synergistic effects of immune-checkpoint inhibitors and hypomethylating agents are currently ongoing and may identify methylation-regulated neoantigens. In the interim, DNA methylation signatures may prove useful for identifying patients likely to respond to therapy and may aid decision-making in treatment of melanoma patients.

## Abbreviations

AJCC: American Joint Committee on Cancer
AKT: RAC-alpha serine/threonine-protein kinase
AML: Acute myeloid leukemia
ASCO: American Society for Clinical Oncology
AZA: Azacitidine
BRAF: v-Raf murine sarcoma viral oncogene homolog B
CIMP: CpG island methylator phenotype
CNS: Central nervous system
CTLA-4: Cytotoxic T lymphocyte-associated protein 4
DAPK: Death-associated protein kinase 1
DEC: Decitabine
DNA: Deoxyribonucleic acid
DNMT: DNA methyltransferase
DSP: Desmoplakin
ECAD: E-cadherin
EMT: Epithelial-to-mesenchymal transition
ERKs: Extracellular-regulated kinases
FRZB: Frizzled-related protein
GNMT: Glycine N-methyltransferase
HCC: Hepatocellular carcinoma
HCK: Hematopoietic cell kinase
HG: Hydroxyglutarate
HIF: Hypoxia-induced factor
HLA: Human leukocyte antigen
HOX: Hoemeobox
IDH: Isocitrate dehydrogenase
IFNG: Interferon gamma
IL: Interleukin
ITK: Interleukin 2-inducible T cell kinase
KG: Ketoglutarate
LATs: Linker for activation of T cells
LEP: Leptin
LINEs: Long interspersed nuclear elements
LXN: Latexin
MAFG: v-Maf avian musculoaponeurotic fibrosarcoma oncogene homolog G
MAGE: Melanoma antigen/melanoma-associated antigen
MAPKs: Mitogen-activated protein kinases
MDS: Myelodysplastic syndrome
MET: Mesenchymal-epithelial transition factor
MGMT: O-6-Methylguanine-DNA methyltransferase
MHC: Major histocompatibility complex
MINT: Msx2-interacting nuclear target
MITF: Microphthalmia-associated transcription factor
MPO: Myeloperoxidase
MSP: Methyl-specific PCR
NRAS: Neuroblastoma RAS viral oncogene homolog
NSCLC: Non-small cell lung cancer
PGRB: Promoter regions B
PSCA: Prostate stem cell antigen
PTEN: Phosphatase and tensin homolog
PTHLH: Parathyroid hormone-like hormone
QPCT: Glutaminyl-peptide cyclotransferase
RAR: Retinoic acid receptor
RICTOR: Raptor independent component of mTOR complex 2
ROC: Receiver operator characteristic
RRBS: Reduced representation bisulfite sequencing
SNP: Single nucleotide polymorphism
SWI/SNF: SWItch/Sucrose Non-Fermentable
SYK: Spleen tyrosine kinase
TCGA: The Cancer Genome Atlas
TERC: Telomerase RNA component
TET: Ten-eleven translocation methylcytosine dioxygenase
TUB: Tubby bipartite transcription factor
WGBS: Whole genome bisulfite sequencing
